# Did Glycopeptide Use in Animals Result in Hospital Infections of VRE?

**DOI:** 10.1371/journal.pmed.0020383

**Published:** 2005-11-29

**Authors:** Anthony Mudd

**Affiliations:** **1**SouthamptonUnited Kingdom

As one of the persons involved with the development of avoparcin for farm animals, I have followed the discussion on vancomycin-resistant enterococci (VRE) and its potential transfer from animals to humans over the past decade. What a pity that the authors of this *PLoS Medicine* Policy Forum [1] did not reference a recent review by Wassenaar [2] that comprehensively discussed this topic. In this review, evidence is presented to show that VRE infections in humans have actually increased in the European Union since avoparcin was removed from the market. Other data show that whole-genome typing methods separate clinical VRE strains from animal or nonhospitalized human strains.

The conclusion of the Smith et al. article [1] that a correct decision was made to adopt the EU “precautionary principle” and remove avoparcin from the market is surprising, as this is contrary to the opinion of the independent EU Scientific Committee for Animal Nutrition, and since a quantitative risk analysis, as suggested by the authors, could not conclude a relationship between glycopeptide use in animals and incidence of clinical infection in humans.
